# Albuminuria in Pregnacy

**Published:** 1889-11-16

**Authors:** 


### ALBUMINURIA IN PREGNANCY.

An interesting case is reported in a recent number of the *Lancet*, by Mr. H. F. Bailey, of Blackheath, of acute epigastric pain in pregnancy, associated with albuminuria. The chief complaint was of pain about the epigastrium, which was attributed to indigestion, but the friends of the woman had noticed that she was unusually "fat" about the face and hands, and the patient herself was aware that the hands and feet were swollen. Mr. Bailey found œdema up to the knees, and a trace of albumen in the urine. At a later period sickness became a marked feature of the case, and the epigastric pain was much increased, shooting through to the back. The mental condition was one of great depression, and the amount of albumen much increased. It was evident the case was one of an unusual and dangerous description, which might necessitate the induction of labour if eclamptic symptoms supervened. While deliberating on a plan of action, premature labour came on. Suddenly the patient was seized with general rigidity, the hands taking the attitude of extreme extension, and the fingers being bent backwards. Chloroform was given, and delivery accomplished by forceps. The mother did well, all albumen and pain disappearing on the second day.

